# One Health (r)Evolution: Learning from the Past to Build a New Future

**DOI:** 10.3390/v10120725

**Published:** 2018-12-18

**Authors:** Ilaria Capua, Giovanni Cattoli

**Affiliations:** 1 One Health Center of Excellence for Research and Training, University of Florida, Gainesville, FL 32611, USA; 2 Animal Production and Health Laboratory, Joint FAO/IAEA Division of Nuclear Technique in Food and Agriculture IAEA, Vienna International Centre, 1400 Vienna, Austria; g.cattoli@iaea.org

**Keywords:** One Health, history, perspective, future, challenge

## Abstract

The One Health concept recognizes that the health of human beings, animals, plants and the environment is interconnected and interdependent. This idea has been shaped over the centuries and has gained momentum and traction as anatomy, physiology, microbiology and other disciplines have substantiated earlier theories. Here we recall major historical milestones which have contributed to shaping the One Health concept as it is today, and discuss the past and future drivers in view of future challenges in an evolving scenario.

## 1. Introduction

The One Health concept which focuses on the connections between the health of humans, animals and the environment, dates back to the ancient world and was compatible with the dominating scientific philosophy of the time, the theory of humours [1]. The interdependency of public health and clean environment was identified as early as Hippocrates (460 BCE–367 BCE) in Airs, Waters and Places, which argued that geographical conditions and climate influence health [2,3]. Many of us are convinced that the Zoobiquity approach is innovative, but as early as 350 BC, Aristoteles (384 BCE–322 BCE) in Historia Animalium presents the first documented approach to comparative medicine [4]. He diversified humans from animals through their possession of a rational soul. However, his intuition led him to relate animals to humans by documenting differences and similarities in the form, function and purpose of their parts. On this basis, he created a taxonomic system. Even Galen of Pergamum (129 CE–2216/217 CE), following Aristotele, wrote extensively about his numerous observations and experiments of animals [5].

Following these lines of thinking, it was assumed at that time, that the prevailing concepts underlying human and animal health were defined by the same physiological system: humouralism, which of course was then proven to be incorrect, but not until after the Middle Ages and well into the Renaissance. Basically, the concept of interconnection and interdependencies between living organisms was correct but the explanation behind it was wrong, simply because they did not have the scientific tools to understand what we know today [6].

A second, very powerful wave of lateral and comparative thinking worked out human anatomy from animals. During the XVIth and XVIIth century, what was described as “comparative anatomy” started to develop with contributions of eminent scientists such as Andreas Vesalius (1514–1564) [7], Realdo Colombo (c. 1515–1559) and Fabricius (1537–1619) who conducted important studies on blood circulation and physiology [8]. Giovanni Maria Lancisi (1654 CE–1720 CE), a pioneering epidemiologist, physician and veterinarian, was the author of papers in which he identified the role the environment plays in the spread of diseases both to human and to animals [9]. The seventeenth and eighteen century movements provided great insight and proved ancient Greek theories were correct in principle by bringing humans and animals into even greater proximity.

In France, Vicq d’Azyr (1749–1794) went beyond comparative anatomy to develop a truly comparative form of medicine. His investigations proved the key role of the environment in human and animal health and diseases. D’Azyr correlated human and animal diseases with climatic and geographical conditions. He did not perceive any dividing line between human and animal medicine [10]. The founding of the first veterinary school in Lyon, France, by Claude Bourgelat (1712–1779) established formal education in animal health and its interaction with human health in Europe. The subsequent work of Louis-René Villermé (1782–1863) and Alexandre Parent-Duchatelet (1790–1835), also in France, led to the development of the veterinary specialty field of public hygiene [11]. It was a little later that the German physician and pathologist Rudolf Virchow (1827–1902) coined the term ‘zoonosis’. He insisted that health in humans and animals differed only in detail and not in kind. He recognized that environmental factors were key determinants of health outcomes. For example, his indications for ending a persistent epidemic of typhus, which he himself had investigated, was to provide the affected region with freedom, improved roads and good schools [12].

The Canadian Sir William Osler (1849–1919), who studied under Virchow’s mentorship, further promoted the concepts of comparative medicine and comparative biology and the integration of human and animal health. He is acclaimed for having coined the term “one medicine” [13], thus considered as the figurehead of One Health. He taught veterinary students, undertook research into the diseases of animals, and once again asserted the value of comparative medicine to medical audiences [14].

The 1860s–1870s marked the development of the germ theory. In France, Louis Pasteur (1822–1895) discovered the principles of vaccination, microbial fermentation, and pasteurization [15].

Robert Heinrich Hermann Koch (1843–1910), the founder of modern bacteriology, identified the specific causative agents of tuberculosis, cholera, and anthrax and provided experimental support for the concept of infectious diseases, which included experiments on humans [16].

## 2. Macrocycles in One Health

From the very brief historical perspective summarized above, it is clear that there are macrocycles of understanding built around contemporary scientific knowledge. The theory of the humours was based on the underlying belief that health is governed by a complex interaction between factors that are both internal and external to the human body. This was an unfounded theoretical hypothesis that landed on scientific evidence only around the 16th century but then started increasing exponentially. An ever-expanding number of scientific discoveries, “milestones of understanding” were developed in animal models and then applied to humans. During the 200 years which separated the studies of Vesalius from those of Pasteur, the One Health approach was rewarded by yielding many game-changing scientific discoveries, mainly in the fields of comparative anatomy, physiology and pathology.

Pasteur, Virchow and Osler were the forerunners of the next macrocycle around One Health—which is still ongoing. Another step forward in knowledge about the interconnection between animal and human health was when zoonotic diseases were discovered and understood in their complexity. These were just part of a much bigger picture; evidence that an animal virus such as rinderpest could give origin to a devastating human virus such as measles was a true revelation [17].

As knowledge progressed, the perspective of Eco-Health as a natural expansion of the germ theory to include ecological aspects was developed. The contribution and inter-dependency of ecosystem health to the health of humans and animals received full recognition in early 2000, when One Health became the dominating approach to emerging threats related to avian and pandemic influenza. This likely triggered the popularity of the One-health concept beyond the limited scientific community, to the point that it was a well-represented issue in the agenda of the International Ministerial Conference on Avian and pandemic influenza in 2008 in Sharm El Sheikh. Since then, successful projects and growing attention have been focusing on the emergence of potentially zoonotic pathogens from wildlife and the ecosystem. Over the past couple of decades, outbreaks of avian influenza, West Nile, Ebola, MERS CoV and many others have urged funding agencies and international organizations to include wildlife in their studies, and even beyond that, some internationally funded projects are targeted to carry out surveillance focused on wildlife [18].

It is clear that One Health is a convincing approach which has evolved over the centuries and is expected to evolve further in light of new discoveries. This special issue was assembled with the intention of collecting diverse experiences and points of view of colleagues who share the One Health methodology in their scientific approach. Such a collection of articles allows us to explore critically the state of the art with reference to the persistence of old challenges and the implementation of successful mitigation strategies.

## 3. Future Perspectives

In looking back at how the One Health vision has developed over the centuries, it seems rather obvious that as we learn more and we develop new ideas, the One Health concept is going to encompass more and more disciplines as new interfaces are discovered and become accessible. As these discoveries occur, the area of overlap between the health of humans, animals and the environment will be in constant expansion. For this reason, One Health should be seen as a dynamic concept evolving over time, taking full advantage of the tools and knowledge available in other fields.

The ever-evolving focus of the One Health approach has brought us today to concentrate on emerging and re-emerging zoonotic infections including those linked to antimicrobial resistance. As guest editors we have stimulated authors to look into novel frontiers of One Health. Several papers contained in this Special Issue highlight a need to look into the broader picture and to explore other drivers and determinants as influencing forces on the implementation of One Health efforts being proposed on a global scale. To do this we must be brave and move towards new frontiers.

The big data environment, the “omics” revolution, social media, and scientific and technological breakthroughs offer great opportunities to expand the frontiers of existing areas of research and to create novel ones. However, this process should also be context driven, dovetailing into higher-level objectives such as the sustainable development goals. The latter offer an opportunity to extend areas of collaboration and research across disciplines. The One Health community should be proactive in the current environment and be more prepared to face contemporary challenges. Artificial intelligence, deep learning, and data driven research will be strategic tools and approaches that will contribute to opening one or more new One Health macrocycles building on previous and ongoing approaches. 

The One Health community needs to remain open-minded and be permeable to (disruptive) ideas, which may point to transformational approaches coming from the contemporary environment including the digital revolution. These must be preceded by repositioning humans to their role as “one of the species inhabiting the planet”, although with great impact, as we can be considered “the species in charge” for the health of the planet.

Above all, the One Health community and its stakeholders should stretch their minds to visualize One Health as a dynamic concept that evolves over time by exploiting knowledge available at that time. The real challenge moving forward will be on how to better understand the areas of interface between the health of humans, animals, plants and the environment and learning to address the health of the system, which is the fundamental concept underlying the One Health approach.
